# Development of a Cancer Treatment with the Concomitant Use of Low-Intensity Ultrasound: Entering the Age of Simultaneous Diagnosis and Treatment

**DOI:** 10.3390/diagnostics4020047

**Published:** 2014-04-22

**Authors:** Makoto Emoto

**Affiliations:** Division of Gynecology, Center of Preventive Medicine, Fukuoka Sanno Hospital, International University of Health and Welfare, 3-6-45, Momochi-hama, Sawaraku, Fukuoka 814-0001, Japan; E-Mail: emoto@kouhoukai.or.jp; Tel: +81-92-832-1100, Fax: +81-92-832-1102

**Keywords:** tumor angiogenesis, color Doppler ultrasound, uterine sarcoma, ultrasound therapy, Sonoporation, anti-angiogenic therapy, VEGF (vascular endothelial growth factor), circulating endothelial cells, low-intensity ultrasound, metronomic chemotherapy

## Abstract

In recent years, studies using ultrasound energy for cancer treatment have advanced, thus revealing the enhancement of drug effects by employing low-intensity ultrasound. Furthermore, anti-angiogenesis against tumors is now attracting attention as a new cancer treatment. Therefore, we focused on the biological effects and the enhancement of drug effects brought by this low-intensity ultrasound energy and reported on the efficacy against a uterine sarcoma model, by implementing the basic studies, for the first time, including the concomitant use of low-intensity ultrasound irradiation, as an expected new antiangiogenic therapy for cancer treatment. Furthermore, we have succeeded in simultaneously utilizing low-intensity ultrasound in both diagnosis and treatment, upon real time evaluation of the anti-tumor effects and anti-angiogenesis effects using color Doppler ultrasound imaging. Although the biological effects of ultrasound have not yet been completely clarified, transient stomas were formed (Sonoporation) in cancer cells irradiated by low-intensity ultrasound and it is believed that the penetration effect of drugs is enhanced due to the drug being more charged inside the cell through these stomas. Furthermore, it has become clear that the concomitant therapy of anti-angiogenesis drugs and low-intensity ultrasound blocks the angiogenic factor VEGF produced by cancer cells, inhibits the induction of circulating endothelial progenitor cells in the bone marrow, and expedites angiogenic inhibitor TSP-1. Based on research achievements in recent years, we predict that the current diagnostic device for color Doppler ultrasound imaging will be improved in the near future, bringing with it the arrival of an age of “low-intensity ultrasound treatment that simultaneously enables diagnosis and treatment of cancer in real time.”

## 1. Introduction

Although Japan is among the countries with the longest life expectancies in the world, we have reached an age in which malignant tumors develop in approximately one of every two people, with approximately one in three people dying of malignant tumors. While Japan is a developed country, the percentage of deaths from cancer is high internationally and we are in a situation which urgently requires further review of anti-cancer strategies and development of new cancer treatments. In recent years, research on the usage of ultrasound for cancer treatment has developed and while high intensity focused ultrasound (HIFU, FUS) has already been clinically applied to several types of cancers, it is not yet considered as an established treatment. On the other hand, due to the development of basic studies in recent years, the enhancement of drug effects by low-intensity ultrasound has been revealed as almost certain. Therefore, we focused on the biological effects and enhancement of drug effects brought by this low-intensity ultrasound energy, implementing basic studies, for the first time, through the concomitant use of low-intensity ultrasound irradiation as an expected new antiangiogenic therapy for cancer treatment. We herein describe this therapy. We would like to recommend the further development of an effective and minimally invasive cancer treatment which simultaneously enables diagnosis and treatment of cancer in real time, by applying low-intensity ultrasound energy.

## 2. Biological Effects of Low-Intensity Ultrasound

**Figure 1:**
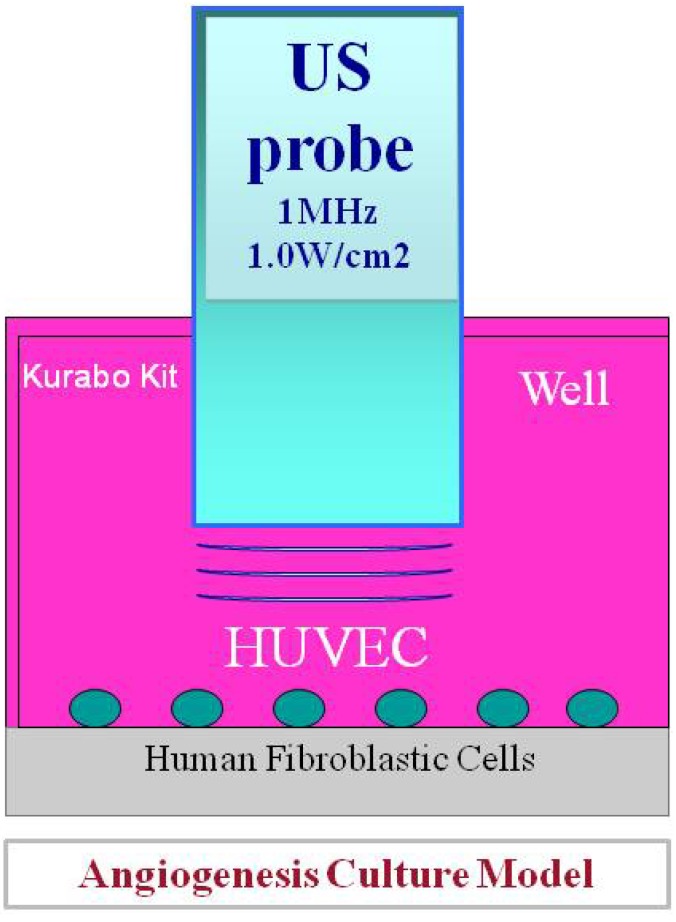
A figure showing a suppression experiment of angiogenesis by ultrasound, designed by the author *et al.* Irradiate ultrasound (using Sonitoron2000: Richmar, USA) per each 1 well under low-output conditions in the state in which human umbilical vascular endothelial cells (HUVEC) and human skin fibroblasts were co-cultivated (KURABO, Japan).

The main biological effects are currently classified into three categories: (1) thermic effect, (2) cavitation effect, (3) non-thermic/non-cavitation effect [1]. Although these effects have not been completely clarified, it is believed that the biological effects of non-thermic and non-cavitation effects occur mainly with low-intensity pulse ultrasound irradiation [2]. In recent years, it has been revealed that transient stomas are formed in cancer cells irradiated by low-intensity ultrasound, a phenomenon known as Sonoporation; acoustic perforation (ultrasound). It is believed that the penetration effect of drugs is enhanced due to the drugs being more charged inside the cell through these stomas. Particularly because cancer cells definitely have more fragile cellular membranes compared with normal cells, the formation of stomas or lacunars is most likely to happen with low-intensity ultrasound [3,4]. Kremkau *et al.* [5] implemented the concomitant use of drug administration and ultrasound irradiation for the first time against leukemia cells in mice and reported on the enhancement effect by drug concentration. Subsequently, the concomitant use of various types of drugs and ultrasound energy has been attempted, with a large number of reports made on the enhancement effect of drugs [6,7,8,9,10]. We experimented with the irradiation of low-intensity ultrasound for the first time for the purpose of developing a new cancer treatment against human umbilical vein endothelial cells (HUVEC) (Figure 1). Furthermore, apoptosis induction by low-intensity ultrasound was clarified, which is a powerful biological effect that can be applied to cancer treatment [10]. Results supporting apoptosis induction were also obtained in our study using a uterine sarcoma model, revealing it as an important biological effect, together with the enhancement effect of drug penetration in our method [9]. However, there are very few reports of molecular-biological studies investigating the mechanism of low-intensity ultrasound treatment, though further investigations are anticipated [11,12]. Investigating how the gene response or gene expression is controlled in low-intensity ultrasound irradiation, using new analysis methods, such as cDNA microarray will therefore be an important step for low-intensity ultrasound treatment in obtaining more scientific grounds [13].

## 3. Anti-Angiogenesis for Cancer and Mobilization Suppression of Circulating Endothelial Cells in the Bone Marrow

**Figure 2:**
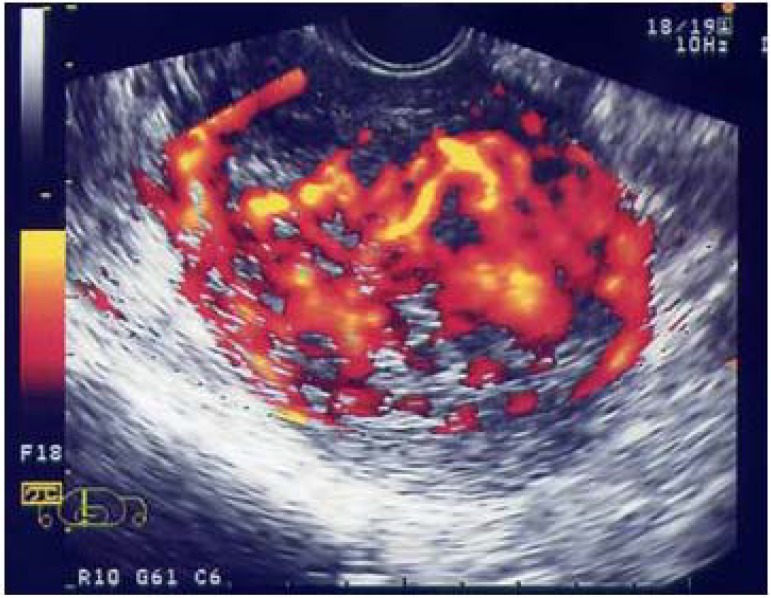
Potential blood flow (angiogenesis) of malignant tumors is enhanced and extracted using color Doppler ultrasound imaging. This picture is a clinical case of uterine sarcoma, using Levovist. Because uterine sarcomas have the most aggressive angiogenesis among all uterine cancers, they are used as a model for angiogenesis.

It has been revealed that angiogenesis plays one of the most important roles in the multiplication of cancer cells and tumor development, which was an epoch-making event in cancer biology in recent years. Furthermore, it has also been discovered that vascular endothelial growth factor (VEGF), in which tumor cells are produced under a low oxygen environment, is the key controlling factor in inducing tumor angiogenesis [14]. Subsequently, the treatment method of starving cancer by controlling angiogenesis has promptly developed further, leading to the clinical approval for the first time in the world of Bevacizumab, an anti-angiogenesis drug, against colorectal cancer. We focused on uterine cancer sarcoma/uterine sarcoma which is an intractable cancer among gynecologic tumors and reported that the angiogenesis of this tumor was extremely intense compared with other uterine cancers (Figure 2) [15,16]. These tumors are extremely malignant among human solid cancers, being a disease with adverse prognosis which shows strong resistance to conventional chemotherapy or radiation therapy. Taking this into consideration, we dared to position this tumor as the subject model of ultrasound treatment and implemented a basic study on angiogenesis suppressive therapy (fumagillin derivative: using TNP-470) by concomitantly using low-intensity ultrasound for the first time [8].

The efficacy of metronomic chemotherapy has been reported as a new cancer treatment in recent years. Metronomic chemotherapy is an approach using frequent dosing of small amounts of anticancer drugs and it has been revealed that anti-angiogenesis is its major action mechanism [17]. Because there is no dosing of large amounts of anticancer drugs at a time in this treatment method, it is advantageous in that it does not lower the immunocompetence of patients, thus leading to anticipation of the concomitant use of ultrasound treatment. Metronomic chemotherapy (irinotecan: CPT-11/SN-38), with concomitant use of low-intensity ultrasound irradiation against a transplant uterine sarcoma model showed significant synergistic effects, revealing that low-intensity ultrasound irradiation enhanced the drug effects in metronomic chemotherapy [9]. In recent years, it has been clarified that circulating endothelial progenitor cells: CEP in the bone marrow are mobilized by VEGF produced by tumor cells or interstitial cells, as a mechanism of enhancing angiogenesis. The fact that the concomitant use of drugs and ultrasonic irradiation synergistically blocked the production of VEGF, which suppressed the induction of CEP cells in mouse bone marrow on the tumor side, was confirmed by the flow cytometry in our study. Furthermore, the apoptosis induction of tumor cells and extension of survival time of cancer bearing mice were found even in the mono-therapy group of ultrasonic irradiation, significantly extending the survival time of the concomitant use group compared with the mono-therapy group [9]. It was also found that an anti-tumor effect was obtained against a uterine sarcoma model, as well as with metronomic chemotherapy, using 5-FU derivative (5'-DFUR) instead of irinotecan [17]. The concomitant use of metronomic chemotherapy and low-intensity ultrasound irradiation against solid cancers is minimally invasive, making it a potential option for ultrasound treatment in the future.

## 4. Proper Drug Dosage Method in Ultrasound Cancer Treatment

The effect of low-intensity pulse ultrasound irradiation can be seen over a short time and frequent implementation over a long period of time. As a result of the investigation in our in vitro study, at IC50 (50% inhibitory concentration) of the ultrasonic irradiation output against human uterine sarcoma cells of FU-MMT-3, IC50 showed 2.27 ± 0.6 W/cm^2^. It was the first basic experiment investigating the IC50 of low-intensity ultrasound against human sarcoma cells and a review of the IC50 for every cancer cell will be necessary in the future. Based on the IC50 in vitro, only the tumor site received radiation (using Sonitoron 2000: Richmar, USA) for 3 minutes, 3 times a week, under the condition of 1MHZ, 50%-DF and 2.0 w/cm^2^ *in vivo.* Almost no adverse events were found in mice, showing good anti-tumor effect [8,9]. Drug administration in concomitant therapy should ideally be implemented frequently in small amounts, prior to ultrasonic irradiation, coinciding with the philosophy of metronomic chemotherapy. In terms of effect enhancement of anticancer drugs, it holds a position similar to that of the philosophy of current chemo-radiotherapy. As its mechanism, it has been revealed that not only by the induction control of CEP cells caused by the production inhibition of VEGF, but also by promoting thrombospondin (TSP)-1, which is an anti-angiogenesis factor produced by stromal tissues, angiogenesis is hindered, enabling the suppression of tumor growth as well [9]. The in vivo model, into which FU-MMT-3 cells [18] that we have established were transplanted, has an extremely high proliferative capacity with tumors, thus making the efficacy of this concomitant therapy very intriguing in consideration of treatment strategies for refractory cancers in the future. In order to design the chemotherapy combined with low-intensity ultrasound irradiation, it is also important to check the half-life of drugs or agents used in this therapy. For example, the half-life of TNP-470, which is a typical anti-angiogenic agent, is short (2–6 min), thus ultrasound irradiation should be performed within a few minutes after the injection of this agent [8]. This study has not only revealed the synergistic effect of low-intensity ultrasound with the concomitant use of drugs, but also turned out to be a study supporting anti-angiogenesis, which was the action mechanism of metronomic chemotherapy.

## 5. Evaluation of the Inhibitory Effect of Angiogenesis by Color Doppler Ultrasound Imaging

**Figure 3:**
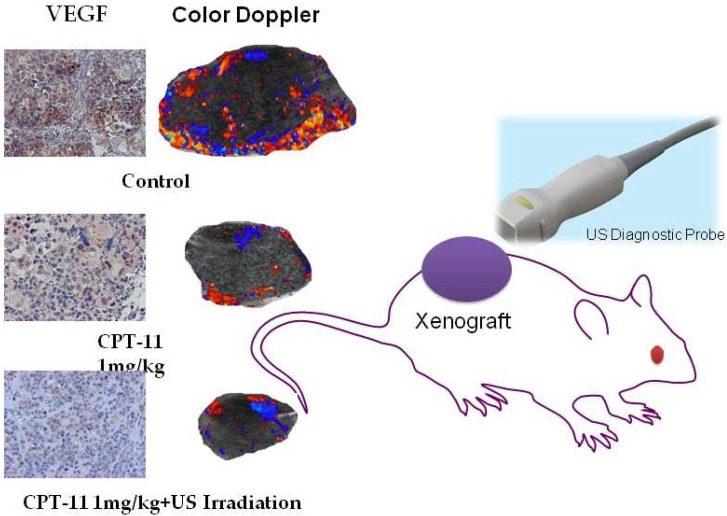
Evaluation of the intratumoral blood flow (angiogenesis) and expression of VEGF by color Doppler imaging: the concomitant therapy of CPT-11 and ultrasonic irradiation causes shrinkage of the tumor volume and significant decrease in intratumoral blood flow, as well as a slight reduction of VEGF.

As clarified in our reports on clinical studies so far, malignant tumors have extremely abundant intratumoral blood flow, compared with benign tumors [19,20,21,22,23]. In this study, not only was ultrasound used for treatment but also low-intensity ultrasound energy was used as the diagnostic tool for effect measurement [8,9]. While tumor volume shrinkage, by image evaluation, is the most important element for the measurement of treatment effect, an additional evaluation of anti-angiogenesis by color Doppler ultrasound imaging provides additional values. Furthermore, the use of ultrasound microbubble contrast agents enables extraction of smaller tumor vessels in real time [24]. The difference in intratumoral blood flow (angiogenesis) by color Doppler imaging among each group was immunohistochemically proven by the chromosomes of VEGF protein and the chromosomes of tumor vascular endothelial cells, using CD31. A significant decrease in tumor vessels and lowered expression of VEGF were found in the concomitant therapy using irinotecan (CPT-11) and ultrasonic irradiation, compared with each monotherapy (Figure 3) [9]. Similar to other studies, it has also been clarified that the contrast effect of blood flow varies depending on the type of ultrasound contrast agent. Although Optison had the highest enhancement effect of intratumoral blood flow compared with Levovist and Sonoview in our early *in vivo* experiments, Sonazoid, which maintains a longer retention time at the tumor site, is currently used [9,17].

## 6. Future of Low-Intensity Ultrasound Treatment: Entering the Age of Simultaneous Diagnosis and Treatment

As our basic study has shown, the simultaneous application of low-intensity ultrasound energy for both diagnosis and treatment will be extremely intriguing in the future development of medical ultrasonics. Because low-intensity ultrasound treatment is instantaneous, efficient and economical, it is expected that this treatment will be a modality that will be fully acceptable in the future medical economy. Taking into consideration future clinical trials, the output of ultrasound energy is almost settable in basic studies so far, with adjustment assumed possible immediately upon modification of the current ultrasound equipment. The development of various types of probes for target diseases will be an issue in the future. The target diseases could be: skin cancer, breast cancer and lymphoma for the body surface; liver cancer and pelvic neoplasm for the abdomen; kidney cancer, prostate cancer and bladder cancer for the urinary organs; and cervical cancer, endometrial cancer, uterine sarcoma, uterine carcinosarcoma for gynecology. It is an urgent task to develop optimal probes suitable for target regions, with the introduction of clinical trials as soon as possible anticipated. Based on the basic studies so far, it is expected that the concomitant use of low-intensity ultrasound and drugs will be able to be applied to the treatment of advanced cancers or recurrent cancers. In our experiments on animals, in which an advanced uterine sarcoma status was set, a significant inhibitory effect on tumors was obtained even though the concomitant therapy was initiated after the tumor grew to a certain extent (late treatment) [9]. Although it is difficult to make tumors disappear completely in advanced cancers, maintaining the status such that tumors do not grow (Tumor Dormancy) might be possible. If equipment is ideally developed, we can expect that the concomitant therapy of low-intensity ultrasound and drugs may be applied even to advanced cancers in which surgical treatment is difficult or recurrent cancers on which radiation therapy or chemotherapy have already been attempted. Furthermore, it is also possible that the new therapeutic chemoembolization that we have developed for the treatment of advanced or recurrent cancers, in which ceramic microspheres are loaded with anti-angiogenesis agents, will make drug delivery more effective through the concomitant use of ultrasonic irradiation [25]. Judging from the standpoint based on the study outcomes so far, the concomitant use of low-intensity ultrasound and small volume of anticancer drugs or anti-angiogenesis agents will be a recommendable approach for cancer-carrying patients with lowered host immunity caused by radiation therapy or chemotherapy. Furthermore, evaluation of tissue elasticity through the concomitant use of elastography will possibly help determine whether or not any tissue changes have occurred due to the ultrasound treatment, such as necrosis.

## 7. In Preparation for Ultrasound Treatment of Uterine Cancer

Currently, an approach using a vaginal probe is commonly used for the ultrasonic diagnosis of pathological changes in the uterus. Though just my personal opinion, in future ultrasound treatments, even without any manufacturing of new treatment equipment, altering current low-power diagnostic equipment to those of beam diagnostic systems may possibly make treatment available for early pathological changes in the uterine cervix. (Author reported at the Special Program “Ultrasound treatment in the field of obstetrics and gynecology: Basic study and clarification of the mechanics of the concomitant therapy of low-intensity ultrasound irradiation in cancer chemotherapy for obstetrics and gynecology” at the 84th Annual Meeting of the Japan Society of Ultrasonics in Medicine). Although surgical resection or panhysterectomy using a laser scalpel is currently common for in situ carcinomas and microinvasive cancer, modifying the current vaginal probe for diagnosis may make it possible to irradiate the uterine cervix within the scope of sufficient ultrasound. This approach is advantageous in that we can preserve the uterus, potentially allowing current invasive surgical therapy to be replaced by future ultrasound treatment. Due to the fact that the growth of uterine sarcomas, which are highly malignant, was suppressed even by a single low-intensity ultrasound irradiation in our experiments on animals, it is believed that it can be clinically applied to the treatment of cervical cancer [8,9].Furthermore, it is expected that the concomitant use of effective drugs could bring about a certain degree of success, even against advanced cancers. In addition, the development of probes of several mm in diameter which can be inserted into the endometrial cavity by modifying the currently widely-used vaginal probe, will also be an interesting approach.

## 8. Drug Delivery System and Microbubbles and Nanobubbles

The concomitant use of low-intensity ultrasound irradiation in drug delivery systems is also useful [25]. Low-intensity ultrasound will become a tool to promote the transport of drugs into cancer tissues, the release of intelligent drugs at the regions reached by these drugs, and absorption into the tissues. The concomitant use of microbubbles or nanobubbles as carriers is expected to bring results that enhance compatibility. Future cancer treatment will develop an effective therapy that is minimally invasive in the human body and bring us a growing expectation of low-intensity ultrasound in this regard as well. We are in the process of developing an ultrasound bubble contrast agent of nanoparticles that is selectively incorporated into the tumor vascular endothelial, using a transplant uterine sarcoma model [26]. We expect further development in the future of ultrasound contrast agents, with a higher detection rate even against much smaller tumor vessels, which are also safer for the human body.

## 9. Conclusions

Recent studies have succeeded in simultaneously utilizing low-intensity ultrasound in both diagnosis and treatment, upon real time evaluation of the anti-tumor effects and anti-angiogenesis effects using color Doppler ultrasound imaging. Based on these achievements, we predict that the current diagnostic device for color Doppler ultrasound imaging will be improved in the near future, bringing with it the arrival of an age of “low-intensity ultrasound treatment that simultaneously enables diagnosis and treatment of cancer in real time.”
